# Dr Ayub Khan Ommaya (1930–2008): The eventful life of a revolutionary neurosurgeon

**DOI:** 10.1177/09677720231198502

**Published:** 2023-09-18

**Authors:** Salim Usman, Sakshi Roy, Arjun Ahluwalia, Muhammad Hamza Shah

**Affiliations:** 1 170904 School of History, Anthropology, Philosophy and Politics, Queen's University Belfast, Belfast, UK; 2 School of Medicine, 1596 Queen's University Belfast, Belfast, UK

**Keywords:** Neurosurgery, history of medicine, Ommaya reservoir, traumatic brain injury, medical devices, south Asian diaspora

## Abstract

Dr Ayub Khan Ommaya (1930–2008) was a pioneering figure in the field of neurosurgery, with a particular focus on traumatic brain injury. As history books have held, he was a man of great intellect and vision, possessing a rare combination of scientific rigour and compassionate empathy. One of Dr Ommaya's most notable contributions was his development of the Ommaya reservoir, a device used to deliver drugs directly into the brain. This groundbreaking technology transformed the treatment of brain tumours and other neurological disorders, enabling clinicians to administer medications with unprecedented precision and efficacy. From his groundbreaking research on traumatic brain injury to his visionary invention of the Ommaya reservoir, Ommaya's legacy continues to inspire and inform the work of countless medical professionals around the world. This historical paper delves into Ommaya's remarkable life story, highlighting his extraordinary contributions to the field of neurosurgery.

## Background

**Figure 1.**
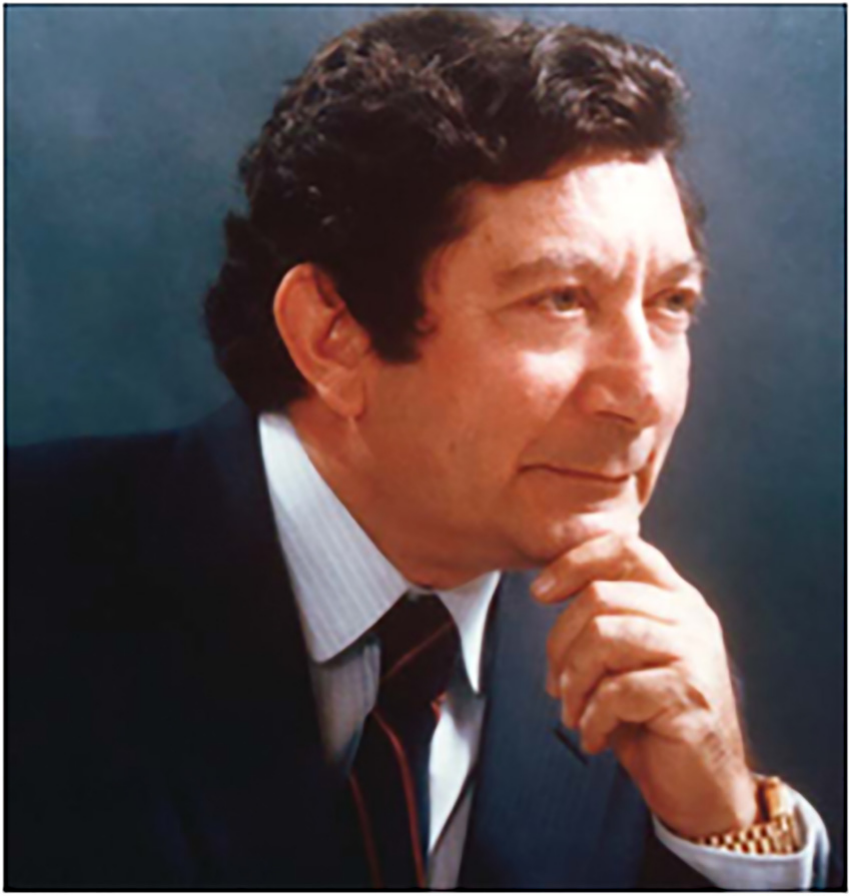
Headshot from Ommaya's obituary published in The Lancet (2008).

Within medical history, there are certain figureswhose contributions to the field stand out as exceptional and elevates them to the status of pioneers. Dr Ommaya, an esteemed physician and researcher (Figure 1),^1^ was one such individual whose remarkable achievements have left an indelible mark on the world of medicine. Born in 1930 in Pakistan, Dr Ommaya received his medical degree from King Edward Medical College in Lahore and went on to obtain a MA from Balliol College, University of Oxford.^2^ Throughout his career he held positions at the National Institute of Neurological Disorders and Stroke, US National Highway Safety Administration and George Washington University, District of Columbia.

Dr Ommaya's career spanned several decades and he made numerous contributions to the field of neurosurgery. He is perhaps best known for his work on the development of the Ommaya reservoir, a device used to administer drugs directly into the brain.^3^ The reservoir consists of a small, dome-shaped container that is surgically implanted under the scalp and connected to a catheter that extends into the brain. The device allows drugs to be delivered directly to the brain, bypassing the blood–brain barrier, and is used to treat a variety of neurological conditions, including tumours, infections, and pain. In addition to his work on the Ommaya reservoir, Dr Ommaya also developed the Ommaya catheter, a tool used to monitor intracranial pressure. The catheter is inserted into the brain through a small hole in the skull and measures the pressure inside the skull, which can help doctors diagnose and treat conditions such as hydrocephalus and traumatic brain injury (TBI).^4^

While at the National Institutes of Health (NIH), Ommaya studied TBI in subhuman primate models and focused his research on understanding the mechanism of cerebral concussions.^5^ He held a special interest in TBI and conducted extensive research on its effects on the brain. Consequently, his studies helped to improve our understanding of the biomechanics of brain injury and the ways in which it can lead to neurological deficits. Later, he also developed a classification system for brain injuries that is still in use across neurosurgical facilities worldwide.

## Early life and career

Ommaya came from humble beginnings in Mian Channu, a small city in Punjab, British India (present-day Pakistan), to Nadir Khan, a British Indian Cavalry member, and Ida, a French Catholic.^2^ At the age of 14, Ayub read a magazine article that piqued his interest in neurosurgery, describing the experiments of Dr Wilder Penfield on the surgical treatment of epilepsy and electrical stimulation of the brain.^2^ Drawing inspiration from Penfield, he decided to pursue a medical career and completed his premedical studies at Gordon College, Rawalpindi, Pakistan, before moving on to King Edward Medical College in Lahore.^2^ At King Edward, he received the Harper-Nelson Gold Medal for outstanding academic achievement and established himself as a champion debater, boxer, and swimmer.^2,6^ At the same time, he also won a regional swimming competition and went on to win the national competition in 1953.

**Figure 2.**
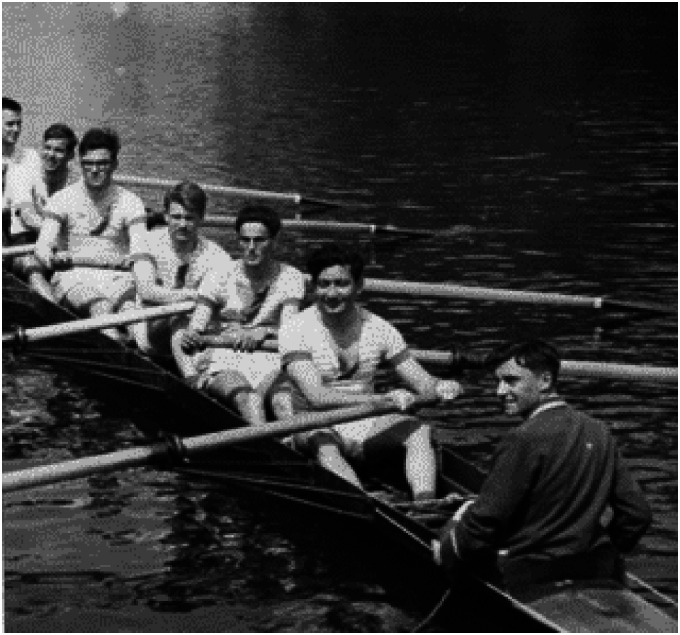
Dr Ommaya participating in a rowing event with his crew team at Oxford University in 1955.

Following his preliminary endeavours, Khan immersed himself in the world of music and studied opera in Venice, Italy, for three weeks.^2^ In Italy, young Khan was fortunate to receive singing lessons from a renowned opera tenor. This experience left a significant imprint on his character and professional life, as he often regaled his colleagues, patients, and their families with his vocal performances before and after surgeries. Subsequently, Khan was honoured with a Rhodes scholarship at Balliol College, Oxford. At Oxford, under the tutelage of the esteemed American neurosurgeon Dr Joseph Pennybacker, he delved deeply into understanding the intricate mechanisms of brain injury. This synthesis of arts and sciences made Khan a unique figure in his field, showcasing the depth and range of his interests and talents. Interestingly, he had initially written to Dr Penfield, asking him to accept him as a student but he wrote back suggesting that Ommaya should consider going to London to study under one of Penfield's old students, Dr Pennybecker.^7^ While at Oxford, he rowed for Balliol (Figure 2)^2^ and won the James Willis Kirkaldy Oxford University Prize in 1956, eventually being offered the prestigious Hunterian Professorship at the Royal College of Surgeons in 1964.

Dr Ayub underwent extensive neurosurgical training under Dr Joseph Pennybacker at Nuffield College of Surgical Sciences and the Radcliffe Infirmary in Oxford. After completing his training, he came to the United States and began working at the Surgical Neurology Branch of the NIH. He was well known for his surgical skill, and his 1977 operation to remove a spinal arterio-venous malformation was highly publicised due to its complexity.^8^ Dr Ayub was also a man of many talents, appearing in the movie Lorenzo's Oil alongside famous actors such as Peter Ustinov, Nick Nolte, and Susan Sarandon.^9^

In the 1960s, he developed the first coma scale at the National Institute of Neurological Disorders and Stroke.^10^ The scale was practical but remained limited to the institute. However, Ommaya's most significant contribution to the field was the development of the Ommaya reservoir, a self-sealing silicone dome with a catheter designed to run through a small hole in the skull to the lateral ventricles of the brain.^11^ This mushroom-shaped reservoir, placed beneath a flap of the scalp, could be topped up with drugs by injection and gentle pressure used to force the drug through the catheter and into the cerebrospinal fluid (CSF). This innovation has had a more lasting impact and has been widely used in treating brain injuries. He also played a pivotal role in developing spinal angiography, which allowed for the visualization of arteries and veins in the spinal cord, leading to a better understanding of spinal cord arteriography.^12^

He published over 150 articles, chapters, and books, including his centripetal theory of TBI. Ommaya also worked on early computed tomography scanning and determined the spatial resolution of the scanner, leading the way for its use in stereotactic surgery.^13^ In addition, he invented the first spinal fluid-driven artificial organ. He also classified non-traumatic CSF rhinorrhea, which had previously been classified only as traumatic or spontaneous, into high-pressure leaks (tumours and hydrocephalus) and normal leaks (congenital abnormalities, focal atrophy, osteomyelitis).^14^ His centripedal theory of TBI identified that the effects always begin at the brain's surface in mild injury and extend inward to affect the diencephalic-mesencephalic core in more severe injury.^15^ In addition, he demonstrated that both translational and rotational acceleration produce focal lesions, but only rotational acceleration produces diffuse axonal injury.

During his time at George Washington University, where he held a chair in neurosurgery from 1980 to 1985, and acted as the chief medical adviser to the US National Highway Traffic Safety Administration during the same period.^16^ He commissioned a report on brain damage from the Institute of Medicine titled Injury in America, which placed the consequences of road traffic accidents and how to avoid them firmly on the medical and political agendas.^17^ This work was instrumental in developing the National Center for Injury Prevention and Control, which received $10 million in funding from the US Centers for Disease Control, focusing on TBI, thanks to a chance encounter with Congressman William Lehman. Ommaya's research and insights changed people's thinking about the mechanism of injury. Faris Bandak, a director of head injury research for the US Department of Transportation and a friend and colleague of Ommaya says, ‘Rotational acceleration has certain effects on the brain that cannot be caused any other way. The understandings of trauma before Ayub's contributions and following them are quite different’.^1^

## Ommaya reservoir

**Figure 3.**
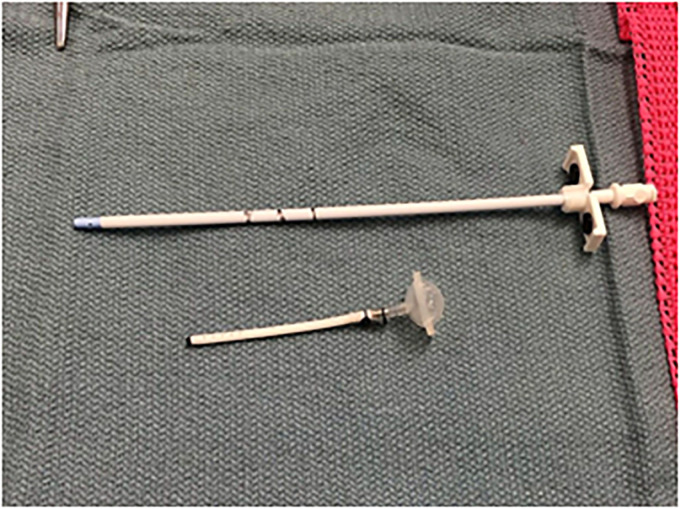
Ommaya Reservoir (affixed to other structures).

Initially designed for the treatment of cryptococcal meningitis through antifungal medication delivery into the CSF,^18^ this device evolved to enable direct CNS chemotherapy administration and CSF extraction without the need for lumbar punctures. The Ommaya reservoir, often referred to as a ‘mushroom-shaped’ device, consists of a capsule and tubing that traverse a small hole in the skull and emerge through the lateral ventricle, positioning itself near the floor of the anterior horn.^3,11^ The capsule's apex, crafted from compressible silicone treated for thickening, facilitates multiple punctures that can self-seal. Beneath the scalp lies the dome of the capsule. Figure 3 provides a visual depiction of the Ommaya reservoir alongside other components securely affixed to it.^19^

The device is implanted by neurosurgeons aided by navigation techniques and once the cutaneous area has healed, these devices do not require special care and may remain in place for months to years while providing controlled access to the ventricular spaces. Haematologists and oncologists frequently utilise these reservoirs for the aforementioned use of introducing chemotherapeutic agents and obtaining CSF samples for dose titration as early as the day of the operation.^3,18,20^ Radiologists also play an essential role in managing these devices, as patients often require imaging to evaluate intracranial disease, tumour staging or, in rare cases, to assess device malfunction. Community nursing staff and general practitioners should be on the lookout for the initial indicators of infection.

In Ommaya's 1962–1968 study involving 60 patients with reservoir implantation for various medical purposes, the primary focus was evaluating the Ommaya reservoir's efficacy and associated morbidity.^21^ A significant 28% complication rate was recorded, leading to surgical interventions for one-fourth of the participants within the first 3 months. Of these, 14 faced device malfunctions, mainly due to tubing issues or tip misplacement. Other complications like CSF culture positivity, bacterial meningitis, and seizures were medically managed. Ommaya noted that as expertise in reservoir placement grew, complications lessened.

## Woodward trial

**Figure 4.**
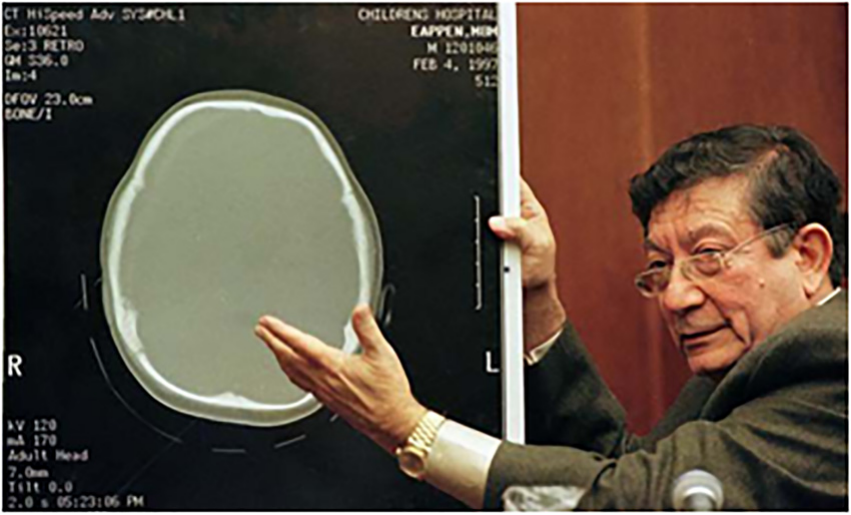
Ommaya giving expert testimony on the Woodward trial in Cambridge, Massachusetts (1997).

In 1997, Dr Ommaya was summoned as an expert witness for the defense in the widely publicised trial of Louise Woodward,^22^ a British au pair who stood accused of causing the death of an 8-month-old infant under her care (Figure 4).^23^ He steadfastly maintained that the child, Matthew Eappen, could not have perished due to violent shaking, as alleged by the prosecutors. During the trial, Ommaya playfully rebounded a lump of Silly Putty on the ground, exemplifying the potential harm resulting from an impact and making his point.^22^

## Affliction for music and later life

Dr Ommaya, often referred to as the ‘singing neurosurgeon’, was not only a trained opera tenor but also an individual deeply entrenched in the Sufi tradition of Islam. He would often sing both before and after surgeries, providing solace to his patients, their families, and his colleagues.^1,2^ His affinity with the Sufi tradition, known for its rich exploration of the inner self and the mysteries of consciousness, profoundly influenced his scientific pursuits. Specifically, the Sufi teachings on the interconnectedness of all existence and the depth of human consciousness provided a philosophical backdrop to his research on TBI. For him, this exploration into the complexities of the human brain was a harmonious blend of spiritual understanding and scientific inquiry. Despite his deep introspection and dedication to his work, he was far from sombre; his approachable demeanour and affable nature endeared him to all, making him not just a respected figure, but a beloved colleague and friend.

Later in his life, Dr Ayub Khan Ommaya made appearances on various television shows, showcasing his expertise, and contributing to public awareness of neurological disorders and their treatments. These appearances allowed him to reach a wider audience and educate people about the advancements in neurosurgery. By appearing on TV shows, Dr Ommaya became a familiar face in the public eye and a respected authority in the field of neurosurgery. His ability to communicate effectively and connect with audiences beyond the medical community further solidified his legacy as a trailblazer in the field.

He was married three times and was survived by his third wife, Ghazala, and six children. Ommaya retired from George Washington University in 2003, and with his dementia progressing, he and his wife returned to Islamabad, where he passed away on 11 July 2008 due to complications of Alzheimer's disease.^1,2,6^ He was well-respected for his surgical skills and received numerous accolades, including the Star of Achievement (*Sitaar-e-Imtiaz*), Pakistan's highest civilian award.^1^ His collaborations with other researchers and clinicians were instrumental to the successful completion of many of his efforts. His legacy lives on, and his work continues to impact the field of neurosurgery to this day.

## Minority trailbrazer

As a person of color as well as a member of the South Asian diaspora, Dr Ayub Ommaya was a pioneer in the true sense of the world, and it's impossible to overstate his importance. Working in a sector often dominated by individuals from the Western hemisphere, his contributions and accomplishments provided not just representation, but also a subaltern perspective to not just neurosurgery, but to the field of health sciences as a whole. His work was groundbreaking in terms of medical advancements as well as in its contributions to inspiring generations of people from Pakistan to choose a career in medicine research. Overcoming prejudice and stereotypes, he emerged as a prominent figure in the field of neurosurgery during a time when representation was sorely lacking. His ability to attain influential positions in academic research was particularly inspiring, considering the challenges he faced.

Moreover, he surmounted cultural and linguistic barriers, as well as the absence of adequate academic and medical infrastructure in his home country – a persistent issue in many nations of the Global South. Despite these obstacles, he rose to become a renowned authority in neurosurgical research. Dr Ommaya shattered the glass ceiling not only for himself but also for the countless Pakistani doctors who currently work abroad, diligently pushing the boundaries of health sciences and saving lives on a daily basis.

Dr Ayub Ommaya's seminal role as a leading figure, as well as his significant contributions to the area of neurosurgery, have had an everlasting effect on the depiction of South Asians in medicine. He challenged deeply ingrained standards, functioning as a catalyst for future generations to overcome institutional barriers and the perceived constraints imposed by the ceiling of racial and class privilege. His notable achievements as a differentiated neurosurgeon and accomplished researcher have not merely resulted in significant advances in medical knowledge but have also inspired individuals of Pakistani and South Asian heritage to pursue careers in medicine and make their own paradigm-shifting contributions. His legacy serves as a source of motivation for the 44 million South Asian individuals currently living abroad,^24^ as well as a guiding force for doctors of colour who push for greater diversity and inclusion within the medical praxis.

## Conclusion

In summary, Dr Ayub Khan Ommaya was an influential figure in the field of neurosurgery, particularly in the study of TBI. His pioneering work on the development of the Ommaya reservoir revolutionised the treatment of brain tumours and other neurological disorders by enabling targeted drug delivery to the brain. Dr Ommaya's research on TBI also provided valuable insights into its mechanisms and led to the development of a widely used classification system. Despite facing challenges as a person of colour and a member of the South Asian diaspora, he overcame prejudice and became a leading authority in neurosurgical research, breaking barriers and inspiring generations of medical professionals. His enduring legacy serves as a source of inspiration for individuals of Pakistani and South Asian heritage, encouraging them to pursue careers in medicine and make their own transformative contributions.
